# Identification of a *SiCL1* gene controlling leaf curling and capsule indehiscence in sesame via cross-population association mapping and genomic variants screening

**DOI:** 10.1186/s12870-018-1503-2

**Published:** 2018-11-22

**Authors:** Haiyang Zhang, Hongmei Miao, Libin Wei, Chun Li, Yinghui Duan, Fangfang Xu, Wenwen Qu, Ruihong Zhao, Ming Ju, Shuxian Chang

**Affiliations:** Henan Sesame Research Center, Henan Academy of Agricultural Sciences, Zhengzhou, 450002 Henan China

**Keywords:** *Sesamum indicum* L., Curly leaf, Indehiscent capsule, Association mapping, SNP/InDel discovery, KAN1

## Abstract

**Background:**

Leaf shape can affect plantlet development and seed yield in sesame. The morphological, histological and genetic analyses of a sesame mutant *cl1* (*cl*) with curly leaf and indehiscent capsule traits were performed in this study. In order to clone the *cl1* gene for breeding selection, genome re-sequencing of the 130 individuals of *cl1* × USA (0)-26 F_2_ population and a bulked segregation analysis (BSA) pool was carried out. The genome re-sequencing data of the 822 germplasm with normal leaf shape were applied.

**Results:**

For *cl1* mutant, the adaxial/abaxial character of the parenchyma cells in the leaf blades is reduced. Results proved that the leaf curling trait is controlled by a recessive gene (*Sicl1*). Cross- population association of the F_2_ population of *cl1* × USA (0)-26 indicated that the target *cl* locus was located on the interval C29 between C29_6522236 and C29_6918901 of *Si*Chr. 1. Further regional genome variants screening determined the 6 candidate variants using genomic variants data of 822 natural germplasm and a BSA pool data. Of which, 5 markers C29_6717525, C29_6721553, C29_6721558, C29_6721563, and C29_6721565 existed in the same gene (C29.460). With the aid of the validation in the test F_2_ population of *cl1* × Yuzhi 11 and natural germplasm, the integrated marker *SiCLInDel1* (C29: 6721553–6721572) was determined as the target marker, and C29.460 was the target gene *SiCL1* in sesame. *SiCL1* is a KAN1 homolog with the full length of 6835 bp. In *cl1*, the 20 nucleic acids (CAGGTAGCTATGTATATGCA) of *SiCLInDel1* marker were mutagenized into 6 nucleic acids (TCTTTG). The deletion led to a frameshift mutation and resulted in the earlier translation termination of the CL gene. The *Sicl1* allele was shortened to 1829 bp. *SiCL1* gene was expressed mainly in the tissues of stem, leaf, bud, capsule and seed.

**Conclusions:**

*SiCL1* encodes a transcription repressor KAN1 protein and controls leaf curling and capsule indehiscence in sesame. The findings provided an example of high-efficient gene cloning in sesame. The *SiCL1* gene and the *cl1* mutant supply the opportunity to explore the development regulation of leaf and capsule, and would improve the new variety breeding with high harvest mechanization adaption in sesame.

**Electronic supplementary material:**

The online version of this article (10.1186/s12870-018-1503-2) contains supplementary material, which is available to authorized users.

## Background

Sesame (*Sesamum indicum* L., 2n = 26) is an important and specific oilseed crop for the high nutrition and oil quality [1]. Sesame is cultivated mainly in the developing countries for its low yield and labor-consuming production process. Therefore, enhancing the yield is one of the key objectives of sesame breeding and production [2, 3]. Sesame yield is affected by many factors, such as plant type, leaf type, capsule number per plant, seed number per capsule, seed size and seed shattering [2, 4–6].

An optimal leaf shape is important to avoid of self-shading and maintains the ideal plant architecture for high yield [7]. Sesame is a kind of high-height crop. Leaf shape can dramatically affect the yield level. A natural sesame mutant (herein named *cl1*) with curly leaves and indehiscent capsules was found in 1940’s [8, 9]. The mutagenesis of *cl1* could affect the plant development and the yield, and the trait is controlled by a regress gene allele [1, 10, 11]. According to the investigation results in 2014 (planted at Pingyu experimental station, China), the *cl1* mutant lines only formed 34–37 capsules per plant. The average seed weight per *cl1* plant was 4.82 g and obviously lower than that (10.17 g) of var. Yuzhi 11 (unpublished data, Haiyang Zhang). To explore the genetic mechanism, Uzun et al. [12] has identified an AFLP (amplified fragment length polymorphisms) molecular marker linked to the mutated trait using BSA (bulked segregation analysis) approach in sesame. However, the target gene for the curly leaf mutation in *cl1* has not been detected mainly for the lack of accurate methods for gene location and validation.

The development of high-throughput sequencing and the initiation of the Sesame Genome Project impel the gene cloning and genomics research in sesame [3]. About one hundred of associated markers and QTLs related to dozens of agronomic traits in sesame have been identified [4, 13–18]. Recently, the gene *SiDt* controlling the inflorescence termination was determined using the ultra-dense SNP map via the fine mapping method [5]. The linkage mapping and GWAS (genome wide association studies) analysis are the two main methods to determine the gene/QTL loci for key agronomic traits in crops. In recent years, integration of linkage mapping and association mapping have been successfully applied in rice [19], cotton [20], rapeseed [21], sunflower [22], soybean [23, 24] for detection of QTLs and candidate genes linked to specific complex agricultural traits. Therefore, in the present study, we sequenced the 130 F_2_ individuals derived from a cross between the curly leaf mutant *cl1* and the wild type, and identified the target gene *SiCL1* alleles in sesame using the cross-population association mapping and the genome variants screening. Meanwhile, the mutagenesis character and the expression profiles of the gene *SiCL1* alleles were systematically analyzed in sesame. The findings supplied the highly-efficient detection method of candidate genes for key agronomic traits and further studies of curly leaf genes in sesame.

## Results

### Characterization of mutant *cl1* and the wild type

**Fig. 1:**
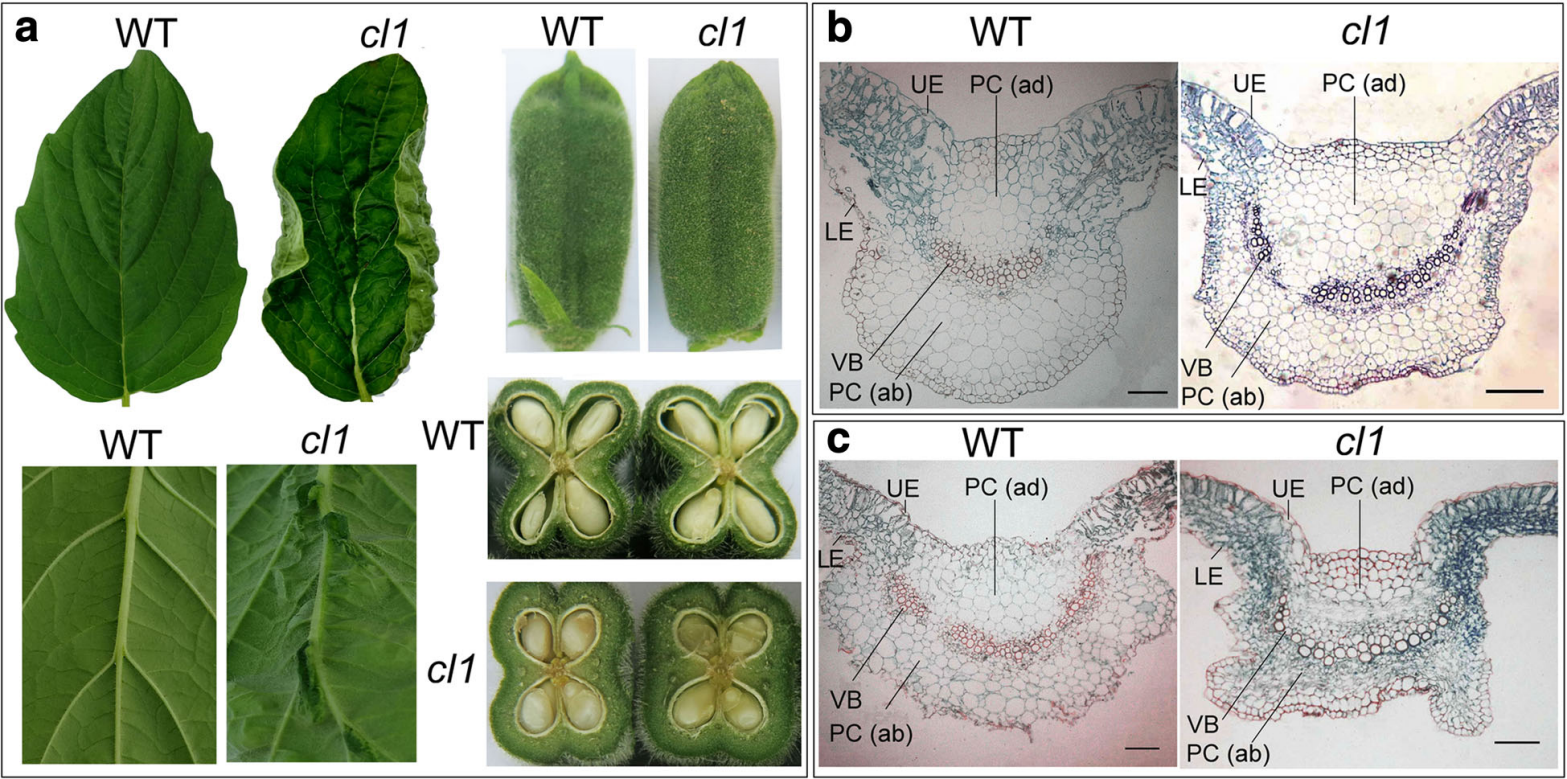
Phenotype comparison of mutant *cl1* and the wild type (WT). **a**: Morphological comparison of leaf and capsule in *cl1* and the wild type (WT, var. Yuzhi 11). In mutant *cl1*, the positive leaf blade is curly, while humps present on the veins of back blade. The capsule tip constrains, and the capsule peel becomes thick. **b**: Cross- section of leaf blades of the one-pair-leaf-old seedlings of *cl1* and Yuzhi 11. **c**: Cross- section of leaf blades of the two-pair-leaf-old seedlings of *cl1* and Yuzhi 11. The PC (ab) and PC (ad) tissues in veins arrange horizontally, and the adaxial/abaxial character is reduced. UE: upper epidemis. LE: lower epidemis. PC (ad): adaxial parenchyma cell. PC (ab): abaxial parenchyma cell. VB: vascular bundle cells. Bar = 100 μm

Systematical phenotypic observation showed that there existed the significant difference in leaf and capsule tissues between *cl1* and the wild type (WT, Yuzhi 11) (Fig. 1). Compared with the wild type (in Fig. 1a), the mutant *cl1* displayed the curly leaf shape character during the entire development life. Some humps presented on the veins of the back blade of leaves. Meanwhile, the tip of capsules was compact and the capsule peel became thick (Fig.1a). After mature or desiccated, the capsules of *cl1* were still indehiscent and the carpels did not split naturally (data not shown). Cross- sections observation of leaf blades indicated that the lower epidemis in *cl1* did not as smooth as that in the WT. The abaxial parenchyma cell (PC (ab)) and adaxial parenchyma cell (PC (ad)) tissues tended to be horizontally arranged in leaf veins. Especially, for two-pair-leaf seedlings of *cl1*, the adaxial/abaxial character was reduced.

### Inheritance of curly leaf trait in mutant *cl1* and the wild type

In order to clarify the inheritance of *cl1* genotype in sesame, we constructed F_1_ hybrids and F_2_ populations of cross combination of ‘Yuzhi 11 ×mutant *cl1*’, and a mapping population of ‘mutant *cl1* ×USA (0)-26’ (Table 1). The F_1_ generations from direct crosses and reciprocal crosses between *CL* and *cl* types only displayed the normal leaf shape with dehiscent capsule. In *cl1* backcrosses of the two combinations, the segregation ratio of the curly and the normal leaf phenotypes fitted the expected 1 (*CL*):1 (*cl*) ratio (Table 1). In addition, the segregation ratio of leaf shape phenotypes in the F_2_ populations fitted the expected ratio of 3 (*CL*):1 (*cl*). Chi-square tests (*P* > 0.05) proved that the segregation of the curly leaf trait in mutant *cl1* fitted the Mendelian inheritance mode, as the χ^2^ value for trait segregation in the above two F_2_ populations was 0.67 and 1.29, respectively. The results confirmed that the leaf curling trait is controlled by a recessive gene allele. Thus, we annotated the curly leaf locus in mutant *cl1* and Yuzhi 11 (WT) as *Sicl1* and *SiCL1*, respectively.

**Table 1:** Inheritance analysis of the *CL* and *cl1* genotypes in sesame

Cross combination		*CL* genotype no. /*cl* genotype no.				Expected ratio	
Parent 1	Parent 2	Direct cross (F_1_)	Reciprocal cross (rF_1_)	BC_1_ (χ^2^ value)	F_2_ (χ^2^ value)	BC_1_	F_2_
Mutant *cl1* (*cl*)	USA (0)-26 (*CL*)	32/0	26/0	221/213 (0.15)	830/261 (0.67)	1:1	3:1
Yuzhi11 (*CL*)	Mutant *cl1* (*cl*)	33/0	29/0	174/165 (0.24)	906/325 (1.29)	1:1	3:1

χ^2^_(0.05, 1)_ = 3.84

### Identification of CL1 gene locus using association mapping and genome variants screening

To efficiently locate the gene locus of curly leaf trait, we carried out the cross-population association mapping and the genome wide screening strategy. The two parents (i.e., USA (0)-26 and *cl1*) and their 130 F_2_ individuals were sequenced using an Illumina sequencing approach. In total, 847.46 Gb of raw data was obtained with the average genome coverage of 18.1 fold per sample (Table 2). The mapped reads of each sample were aligned to the updated sesame reference genome (based on GCA_001692995.1 version) for SNP calling. A total of 598,322 variants were detected between the two parents.

**Table 2:** Genome sequencing information of the mapping population for curly leaf trait

Sample name	Raw read number	Coverage (×)^a^	Ratio of high-quality reads		Variant loci number	Unique Loci^c^
			Q ≥ 20	Q ≥ 30		
Mutant *cl1* (*cl*) (P_1_)	55,244,276	23.41	95.82	91.27	502,626	249,563
USA (0)-26 (*CL*) (P_2_)	54,871,226	23.25	96.30	92.05	601,822	348,759
130 F_2_ progeny^b^	5,539,666,734	2347.32	96.27	91.96	/	/
Total	5,649,782,236	2393.98	96.27	91.96	/	/

^a^The genome coverage is calculated based on the sesame genome size of 354 Mb estimated by K-mer [3]

^b^For the genome sequences of 130 F_2_ progeny, the genome coverage per progeny is 18.06 fold

^c^Unique variants in a parent after compared with the other parent

**Fig. 2:**
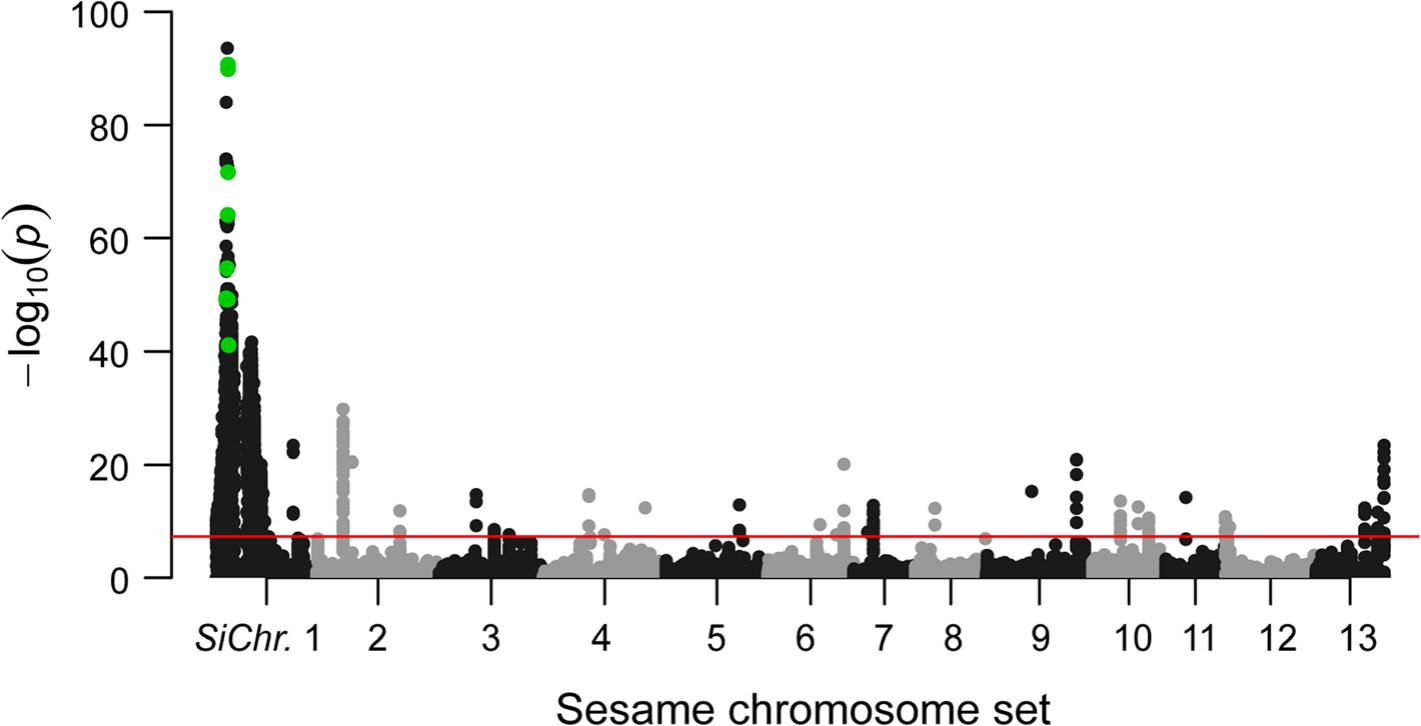
Genomic association mapping of *Sicl1* gene locus in sesame. Mahattan plot of SNP/InDel association analysis is performed using the F_2_ population data. An interval in *Si*Chr. 1 shows the lowest *P* value. All the variants in the plot are screened using the genome variants data. The 6 green dots are retained as the candidate markers linked to the curly leaf trait

The joint calling indicated that 425,661 SNP/InDel variants were located on 13 chromosomes (Fig. 2). The cross-population association mapping showed that an interval on LG 8 displayed the lowest *P* value. Based on the specific variant with the lowest *P* value (C29_6721563, 2.38 × E^− 91^), up- and down-stream 200 Kb flanking sequences of C29_6721563 was determined as the target interval linked to the curly leaf shape (*cl*). The interval C29 between C29_6522236 and C29_6918901 markers contained 90 variants with the *P* value variation of 3.97 × E^− 19^ to 3.00 × E^− 94^ (Additional file 1: Table S1). According to the interval site of Scaffold C29 and the chromosome nomination described by Zhao et al. [25, 26], the LG 8 of the cross population corresponded to the *Si*Chr. 1 of *S. indicum*.

To screen the target SNP/InDel marker, we filtered the located SNP/InDel variants in the interval C29 using the regional genome variants data of 822 sesame accessions (M1-M822) (Additional file 2: Table S2) (Fig. 2). The results showed that 6 variant loci (the green dots in Fig. 2) were retained in the population. At the same time, we screened the plotted variant sites using the BSA pool data for *cl1* (*cl*). The reads of the BSA pool was 38.78 Gb (deposited in NCBI dataset). As a result, 42 SNP/InDel sites were still retained in the interval C29 (Additional file 3: Table S3).

Subsequently, we compared the above two groups of the screening results (shown in Additional file 1: Table S1 and Additional file 3: Table S3, respectively). Interestingly, we found that the 6 variants screened using the variants data of 822 germplasm existed in the 42 candidate variants filtered through the BSA pool data. Of the 6 variants, 5 sites belonged to InDel mutation type (Additional file 4: Table S4). C29_6674693 was located in the intergenic region, while the other five sites of C29_6717525, C29_6,721,553, C29_6721558, C29_6721563, and C29_6721565 were individually distributed in the 5′ flanking, exon or intron regions of C29.460. Therefore, based on the integrated sequences of the four markers (C29_6721553, C29_6721558, C29_6721563, and C29_6721565) in gene C29.460, we designed the primer pair of *SiCLInDel 1* and performed the verification of these candidate markers using the test population (Additional file 5: Table S5). As a result, the *SiCLInDel 1* allele of gene C29.460 entirely accorded with the phenotype segregation in the 1000 F_2_ individuals and the phenotype of the 500 sesame germplasm materials with normal leaf shape (partially shown in Additional file 6: Figure S1). Therefore, C29.460 was regarded as the candidate *CL* gene (i. e., *SiCL1*) for the curly leaf shape in sesame.

### Structure analysis of *SiCL1* gene and SiCL1 homolog in sesame

**Fig. 3:**
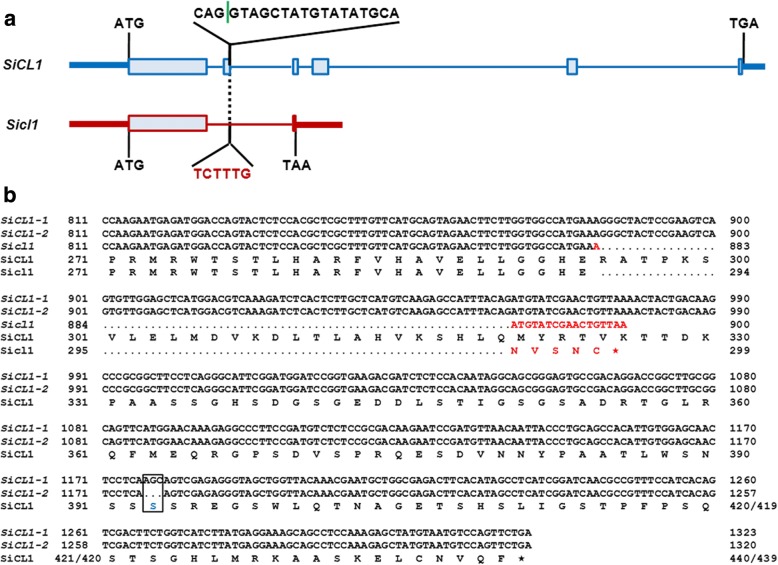
Sequence comparison of *SiCL1* alleles in sesame. **a**: Gene structure comparison of *SiCL1* and *Sicl1*. In the second exon, 20 nucleic acids (CAGGTAGCTATGTATATGCA, 1131–1150 bp in *SiCL1*) are mutated into 6 nucleic acids (TCTTTG, 1131–1136 bp in *Sicl1*). Blue line refers to *SiCL*1 gene. Red line refers to *Sicl1* gene. Green vertical line indicates a splicing site. **b**: cDNA sequence alignment of *SiCL1–1*, *SiCL1–2* and *Sicl1* alleles and the corresponding amino acids. The dots after the red letter ‘A’ in *Sicl1* gene sequences refer to the spliced fragment. The red letters in Sicl1 protein sequence refer the new amino acids formed by the frameshift mutation. The red asterisk refers to the terminator. The blocked sequences in *SiCL1–1* and *SiCL1–2* refer to a splicing site

With the aid of the reference genome information, we designed the primer pairs and amplified the entire cDNA and DNA sequences of *SiCL1* (C29.460) (Fig. 3) (Additional file 5: Table S5, Additional file 7: Table S6). Sanger sequencing and gene alignment results proved that the full length of *SiCL1* in Yuzhi 11 is 6835 bp and comprised 6 exons and 5 introns (Fig. 3a). During sequencing, we found that the *SiCL1* gene had two transcripts of *SiCL1–1* and *SiCL-2*, respectively. *SiCL1–1* encoded 440 amino acids, while *SiCL*-2 encoded 439 amino acids for the deletion of one amino acid (Ser_393_) (Fig. 3b). Structure analysis validated the presence of a splicing site at the position of 4886- 4888 bp (NCBI accession no. MG763174).

In mutant *cl1*, the 20 nucleic acids of CAGGTAGCTATGTATATGCA (1131- 1150 bp) *SiCLInDel 1* marker adjacent to the 2nd exon region of C29.460 allele were mutagenized into 6 nucleic acids of TCTTTG. Interestingly, one splicing site (at the position 1134 bp) existed in the above 20 nucleic acids of *SiCL1* allele. The sequence deletion mutation resulted in a series of change in gene structure as follows (Fig. 3b). With the deletion of the *SiCLInDel 1* marker and the splicing site, a frameshift mutation formed at the position 883 bp of the cDNA of *Sicl1*, and led to the substitution of the five amino acids from Arg_295_-Lys_299_ to Asn_295_-Cys_299_ during translation. Consequently, the new terminator TAA substituted the serine at the residue 300 and resulted in the translation termination (Fig. 3a, b). Then the gene length of *Sicl1* shortened to 1829 bp. The mRNA was 900 bp and only encoded 299 amino acids (Fig. 3b). Of note, a conserved MYB domain (Pro_271_-Thr_323_) was detected in SiCL1. The above frameshift mutation and the terminator formation coincidentally occurred in the conserved region.

**Fig. 4:**
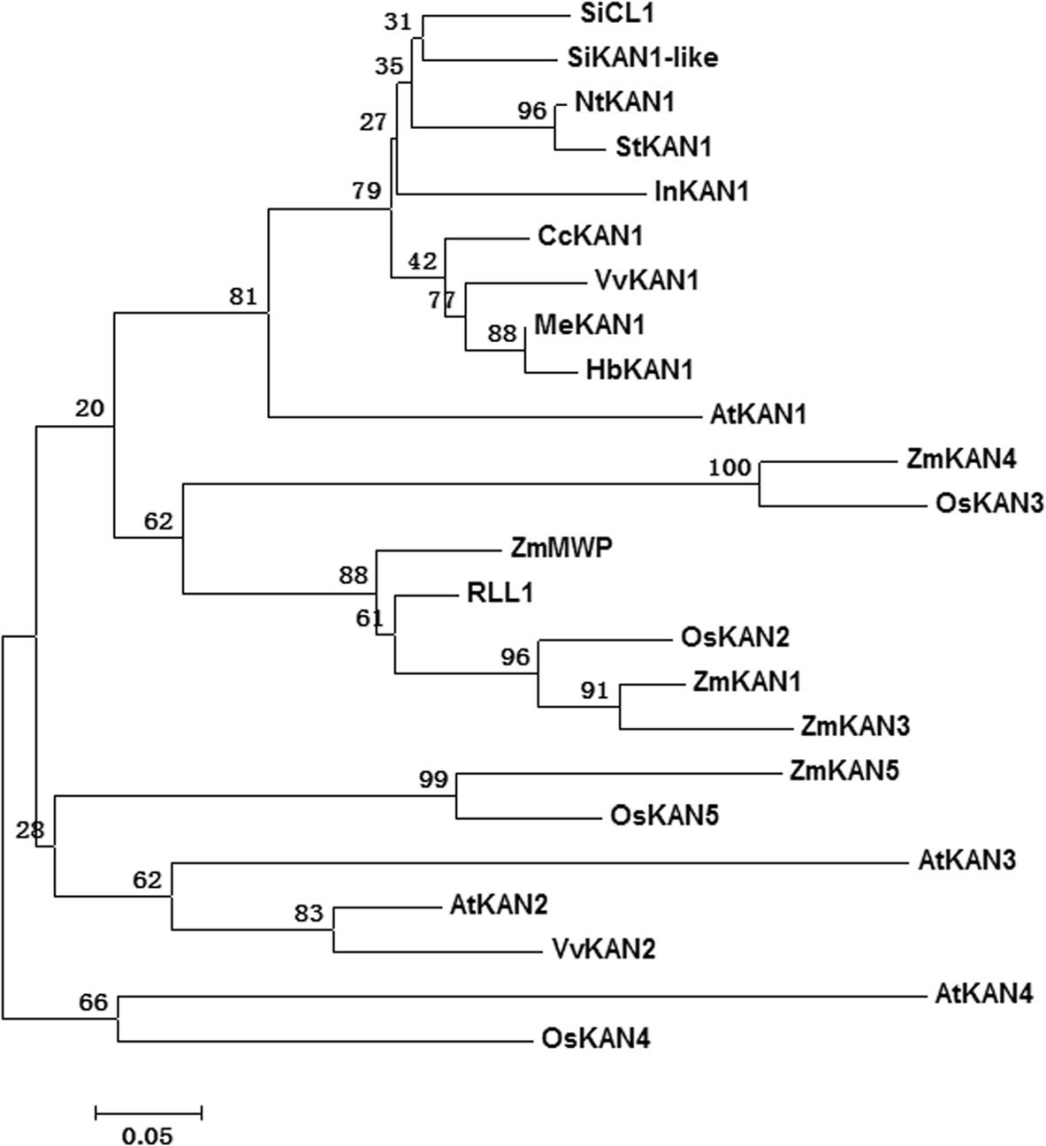
Protein sequence comparison of SiCL1 and KAN homologs in sesame and other four plant species. The blocked region represents the highly conserved MYB domain in members of the GARP gene family. The proteins includes SiCL1 protein and 15 homologs in ten plants, i.e., SiKAN1-like (XP_011099920.1) in *Sesamum indicum*, AtKAN1 (AAL05436.1), AtKAN2 (AAL05437.1), AtKAN3 (AAL05438.1) and AtKAN4 (AAL05439.1) in *Arabidopsis thaliana*; ZmMWP (NP_001147322.1), ZmKAN1 (NP_001295437.1), ZmKAN3 (NP_001307939.1), ZmKAN4 (XP_008661896.1) and ZmKAN5 (NP_001132886.1) in *Zea mays*; RLL1 (XP_015610960.1), OsKAN2 (XP_015648273.1), OsKAN3 (XP_015648944.1), OsKAN4 (XP_015629095.1) and OsKAN5 (XP_015623607.1) in *Oryza sativa*. Identical residues are shaded in black; conserved residues are shaded in gray; residues with low identity are shaded in light gray. The black dot indicates amino acid gap

**Fig. 5:**
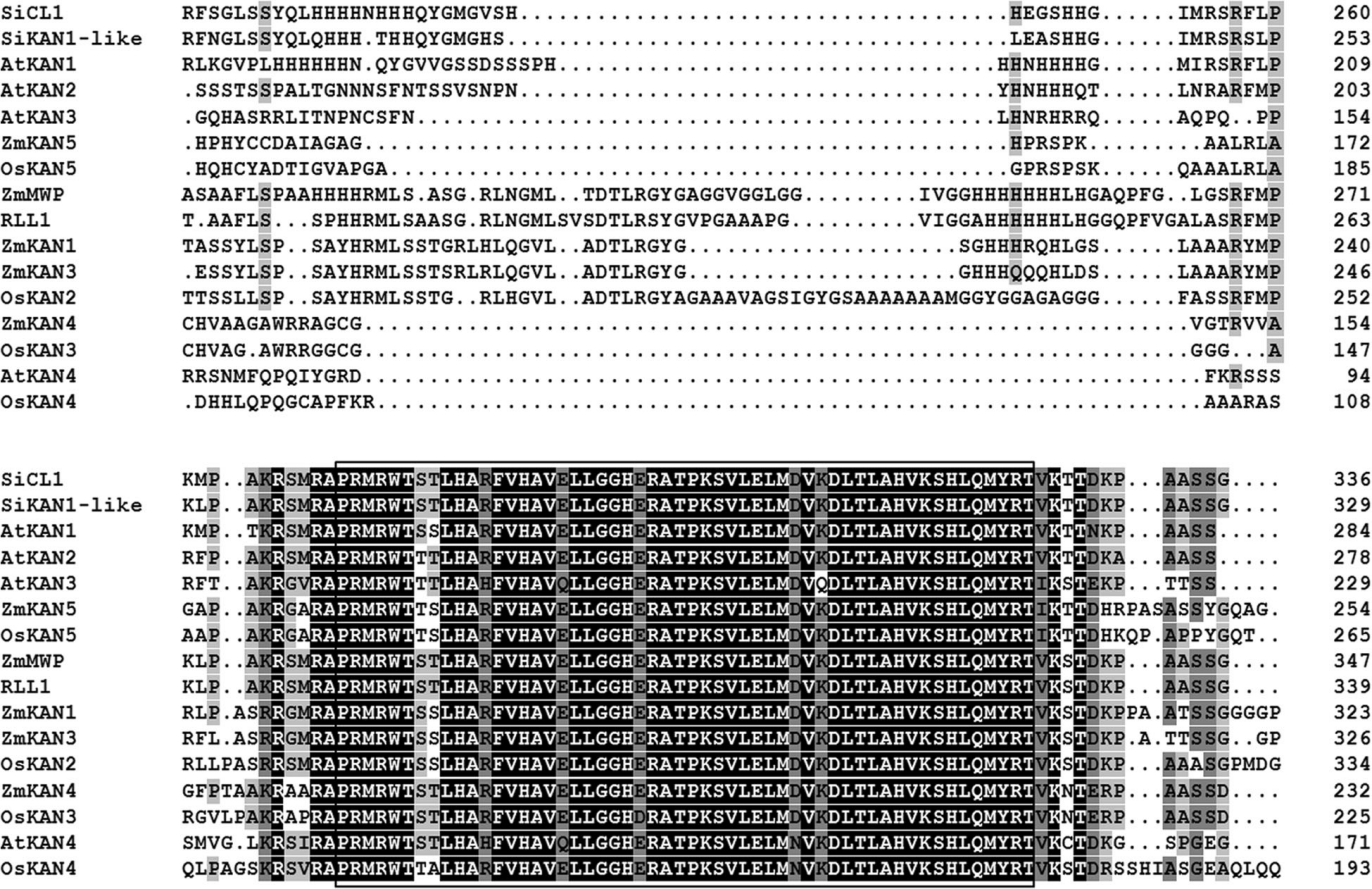
Phylogeny analyses of SiCL1 protein and KANAD1 family in sesame and other ten plants. Numbers above the branches indicate the support with 1000 bootstrap replications. The cluster includes SiCL1 protein and 23 homologs in ten plants, i.e., SiKAN1-like (XP_011099920.1) in *Sesamum indicum*, AtCL1 (AAL05436.1), AtKAN2 (AAL05437.1), AtKAN3 (AAL05438.1) and AtKAN4 (AAL05439.1) in *Arabidopsis thaliana*; ZmMWP (NP_001147322.1), ZmCL1 (NP_001295437.1), ZmKAN3 (NP_001307939.1), ZmKAN4 (XP_008661896.1) and ZmKAN5 (NP_001132886.1) in *Zea mays*; RLL1 (XP_015610960.1), OsKAN2 (XP_015648273.1), OsKAN3 (XP_015648944.1), OsKAN4 (XP_015629095.1) and OsKAN5 (XP_015623607.1) in *Oryza sativa*; MeCL1 (XP_021601546.1) in *Manihot esculenta*; NtCL1 (XP_016437587.1) in *Nicotiana tabacum*; HbCL1 (XP_021642823.1) *of Hevea brasiliensis*; InCL1 (XP_019172029.1) in *Ipomoea nil*; CcCL1 (CDP01671.1) in *Coffea canephora*; and StCL1 (XP_015166410.1) in *Solanum tuberosum*

Non-redundant (NR) protein annotation indicated that the SiCL1 protein belonged to a transcription repressor KAN1 protein. In the sesame genome, there was a homolog gene (named *SiKAN1-like*) to *SiCL1* with the gene resemblance of 70.1% (data not shown). Interspecies comparative analyses indicated that SiCL1 had the high resemblance with the transcription repressor KAN1/ KAN1-like protein in other ten plants (Fig. 4). The resemblance rate varied from 32.8% (ZmKAN1) to 70.1% (SiKAN1-like). The conservative MYB domain existed in these *SiCL1* homologs. Of the 22 homologs in the other 10 plants, KAN1 homologs of potato (*Solanum tuberosum*) and tobacco (*Coffea canephora*) displayed the closer relationship with SiCL1 protein (Fig. 5).

### Expression profiles of *SiCL1* alleles in sesame

**Fig. 6:**
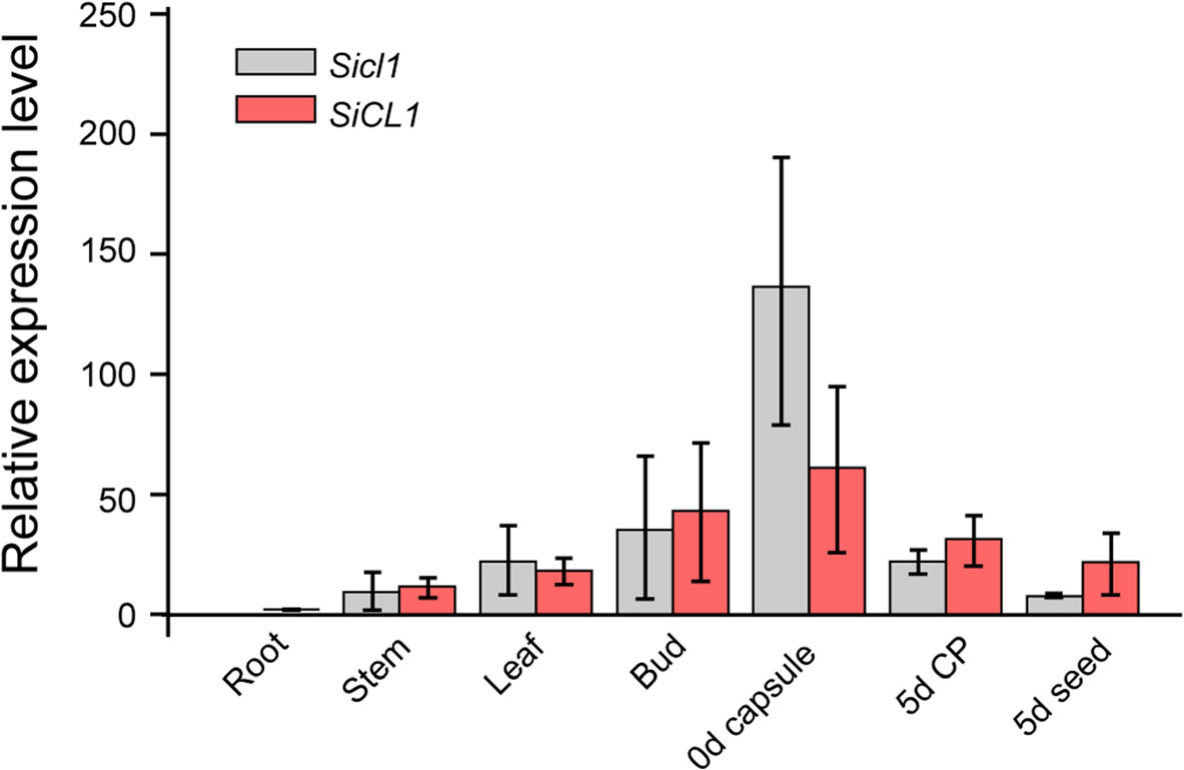
Expression profiles of *SiCL1* gene alleles in the wild type and *cl1*, respectively. The tissues of root, stem, leaf, bud, 0d capsule, 5d capsule peel (CP) and 5d seed are collected from Yuzhi 11 (WT) and *cl1*, and measured with three biological replication

To explore the expression profiles of *SiCL1* and *Sicl1* in sesame, we monitored and compared the transcription level of *SiCL1* alleles in root, leaf, stem, bud and capsule tissues of the WT (Yuzhi 11) and mutant *cl1*, respectively ((Fig. 6) using qPCR assay. In Yuzhi 11, *SiCL1* gene was expressed in root, leaf, stem, bud and capsule tissues. The maximum and the minimum amounts of *CL1* transcript presented in 0 DAP (day after pollination) capsule and root, respectively. The transcript amount in 0d capsule was 61.04 times than that of root, followed was the bud sample (43.68 times). Similarly, the relatively high expression level of *Sicl1* gene was observed in bud and 0 DAP (day after pollination) capsules. The amount of the *Sicl1* transcript reached 136.04 times than that of the root sample.

## Discussion

Leaf shape takes the function of capturing light energy and synthesizing organic compounds through photosynthesis and finally affects the seed yield in sesame [2, 7]. To our knowledge, there are more than six leaf curling types (such as gentle curling, up-curling, up-curling with indehiscent capsule (*cl1* type), curling and shrinking, down-curling, and whole curling) found in sesame mutant library [27] (Haiyang Zhang, the Sesame Genome, ISBN 978–3–319-98098-0, in press). In this study, the morphological and genetic analyses of mutant *cl1* were systematically performed. The target gene *SiCL1* controlling the leaf shape and capsule indehiscence was cloned in sesame. Compared with the normal sesame varieties, *cl1* mutant presented the lower yield level [1, 10, 11]. Meanwhile, the leaf curling trait of *cl1* mutant was synchronized with the capsule indehiscence. Even though the cl1 mutant presents the negative effect of *cl1* gene on the growth and development and seed yield in sesame, the mutation characteristics and the *SiCL1* gene supply the opportunity to explore the development regulation of leaf and capsule, and would impel the harvest mechanization in sesame. Meanwhile, the integrity strategy of cross-population association mapping and the regional genome variants screening provided an example of high-efficient gene cloning in sesame for the first time.

Traditionally, candidate genes/QTLs linked to the specific trait are identified using mapping populations in crops [26, 28, 29]. Recently, combined linkage- association mapping strategies have been applied for detecting the genes or QTLs of quantitative traits in crops [30, 31]. In sesame, the first ultra-dense SNP genetic map was applied for the cloning of *SiDt* gene [5]. About 30,193 SNPs were located in the 13 linkage groups of the SNP map. The main objective of constructing this SNP map with high-resolution markers was to assist in the fine genome assembly and the genomics analysis in sesame. Nevertheless, constructing an ultra-dense linkage map was time-consuming and lavish to clone a functional gene of a quality trait. In this paper, we carried out the common genome joint calling in the 130 individuals of the F_2_ population. A total of 425,697,661 variants were directly plotted in the 13 chromosomes (Fig. 2). Based on the accurate association analysis of the variants, an ~ 400 Kb physical region with significantly high *P* value was determined in linkage group 8. Compared with the traditional linkage mapping method, cross-population association mapping analysis is fast and efficient, as the procedures of constructing a genetic map could be ignored [31]. The association analysis of all the variants with phenotype information in the mapping population could directly performed using GLM (general linear model) model in TASSELE5.0. In addition, candidate-gene analyses within linked regions have been greatly accelerated by the availability of the complete sequence of the genome [32]. To accurately carry out the association mapping, we used the fine sesame genome (Yuzhi 11, GCA_001692995.1), SNP genetic map [5], and the chromosome set annotation system described by Zhao et al. [25] in this study. The high-quality genome supplied the reliable genomic information.

As to the genome screening procedure, we applied both the genome variants data of sesame germplasm and the variants data of a BSA pool to screen the candidate variants in the interval C29. The genome variants data of sesame germplasm contained 8,837,186 SNP/InDel variants (data not shown) and was applied in this study. As shown in Additional file 1: Table S1 and Additional file 4: Table S4, 84 of 90 variants in C29 were filtered. However, for the BSA with 50 individuals with *cl1* genotype, the 191,008 bp distance of the interval between C29_6721553 and C29_6912561 markers presented the significant linkage (Additional file 3: Table S3). For screening the target variant in the tight linkage region, genome variants data is more useful than BSA pool data.

Previous studies showed that leaf curling or rolling was affected by the polarity of cells and complicated development controls [33–35]. Three gene families, i.e., HD-ZIP III family, KANAD1 family and YABBY family are found to regulate the establishment of polarity of leaf in plants [35]. For example, the leaf rolling gene SHALLOT-LIKE1 (SLL1)/RL9 in rice encodes a transcription factor of KANADI family and regulates the leaf abaxial cell development [36]. In this study, cross-section observation reflected that the polarity of the abaxial parenchyma cells (PC (ab)) and adaxial parenchyma cell (PC (ad)) was affected in *cl1* (Fig. 1). The phenotype of leaf curling indeed accorded with the function of the target gene *SiCL1* in sesame. Analysis results reflected that SiCL1 belonged to the KAN1/ KAN1-like protein family, and was the closest to CcKAN1 (CDP01671.1) (Figs. 4 and 5). Similar to the KAN homologs, SiCL1 contained the conserved MYB domain in GARP family (Figs. 3 and 5). As the conserved MYB region was mutated, the gene lost the polarity control. Therefore, we inferred that the frameshift mutation and translation termination resulted in the function loss of *SiCL* in sesame. The results supplied the foundation for elucidating the regulation of the molecular mechanism on leaf curling and capsule indehiscence traits in sesame.

## Conclusions

For *cl1* sesame mutant, curling leaf and indehiscent capsule traits are controlled by a same recessive gene (*Sicl1*). The adaxial/abaxial character of the parenchyma cells in the leaf blades is reduced. The target gene *SiCL1* is a transcription repressor KAN1 homolog with the full length of 6835 bp. The 20 nucleic acids (CAGGTAGCTATGTATATGCA) were mutagenized into 6 nucleic acids (TCTTTG) and eternally resulted in a frameshift mutation and earlier translation termination of the CL gene in *cl1*. The findings provided an example of high-efficient gene cloning in sesame and supply the opportunity to explore the development regulation of leaf and capsule and to improve the new sesame variety breeding with high harvest mechanization adaption.

## Methods

### Plant materials and cross populations

A mutant *cl1* with leaf curling and capsule indehiscence traits was a kind of gift from Mr. Ray Langham, USA in 1980’s. A line (N49) of mutant *cl1* with the curly leaf phenotype was chosen and self-pollinated more than six generations before the genetic analysis. Yuzhi 11, USA (0)-26, and 500 germplasm accessions (M1-M500) with normal leaf shape (listed in Additional file 2: Table S2) were randomly chosen from the sesame germplasm bank to investigate the inheritance of the curly leaf and indehiscent capsule traits in sesame.

The mutant *cl1* and the wild type (Yuzhi 11) were cultured at the Yuanyang experiment station for trait investigation and histological analysis during 2012–2014. Two cross combinations of *cl1* (*cl*, P_1_) and USA (0)-26 (*CL*, P_2_), and Yuzhi 11 (*CL*, P_1_) and *cl1* (*cl*, P_2_), respectively, were cultured for phenotypic segregation analysis in 2012 and 2013 (Table 1).

The 130 F_2_ individuals derived from the cross between *cl1* and USA (0)-26 and the two parents were chosen for genome re-sequencing and gene locus detection. A total of 1231 F_2_ individuals of the cross between *cl1* and Yuzhi 11 and 500 natural sesame germplasm (M1-M500) were cultured at Sanya experimental station for the target gene validation. Fifty individuals of *cl* genotype were chosen from the test F_2_ population and were sequenced as a BSA pool. All the above materials were available from Henan Sesame Research Center, Henan Academy of Agricultural Sciences (HSRC, HAAS) (Zhengzhou, China).

The leaf shape and capsule indehiscence traits in each sample were assessed during seedling, flowering and (or) maturation stages. Chi-square tests (*P* = 0.05) were used to determine the segregation significance for the curly leaf trait. Young leaf tissues of the above accessions and population progeny were collected, immersed in liquid nitrogen and frozen at − 70 °C for genomic DNA extraction. To clone the cDNA sequences and assay the expression pattern of *SiCL1* alleles, the young roots, stem segments, young leaves, buds, 0d capsules and 5d capsules of the *cl1* line N49 and Yuzhi 11 (wild type) at flowering stage were harvested and immersed in liquid nitrogen for RNA extraction.

### Histological analysis of mutant *cl1* and the wild type

Before producing paraffin sections, the fresh of leaf tissues of *CL* and *cl* genotypes were fixed in formalin-glacial acetic acid-alcohol (FAA) solution containing 3.8% formalin, glacial acetic acid and 70% alcohol (V:V:V = 1:1:18) for 24 h, and then air-extracted using pump. The samples were dehydrated with a series of ethanol and stained with 1% safranin for 24 h. After infiltrated using a series of ethanol and dimethybenzene solution for 5 h, the samples were immersed in a series of dimethybenzene solution for overnight. After embedded in paraffin, cross section (10 μm) were cut and stained with 0.1% fast green. The specimens were observed and photographed under the Leica DM6000B microscope equipped a DFC500 camera (Leica, Germany). Image quality was optimized in Adobe Photoshop 7.0 (Adobe, USA).

### Genomic DNA extraction, library construction and sequencing

The two parents (i.e., USA (0)-26 and *cl1*) and their 130 F_2_ individuals and a BSA pool were sequenced using an Illumina HiSeq 2500 sequencing approach. Genomic DNA was extracted from young leaves of each sample using DNeasy Plant Mini Kits (QIAGEN, Hilden, Germany) and fragmented by sonication. Standard paired-end (PE) libraries were constructed according to Illumina guidelines. The libraries were prepared and sequenced on an Illumina HiSeq 2500 platform (Illumina, San Diego, USA) according to the procedures described by Zhang et al. [5].

### Sequencing data analysis and variants detection in population

Raw reads obtained from the Illumina HiSeq 2500 platform were filtered using Trimmomatic 0.33 [37]. Based on the ultra-dense SNP genetic map [5] and chromosome annotation results [25], the genome data of Yuzhi 11 (PRJNA315784) (version 2.0) was re-assembled and applied as the reference genome in this study [38]. Alignment of re-sequencing data to the reference genome was performed using BWA 0.7.15 with the default settings described by Li and Durbin [39]. Putative SNPs and InDels were screened using Genome Analysis Tool Kit (GATK3.7) packages according to GATK joint calling best practice [40].

All the variants from all the 132 sequencing samples were filtered according to the following high quality (high-confidence) criterion: minimal variant count ≥100, minimum frequency of 0.1 (the minimum frequency of the minority polymorphisms for the site).

### Statistical significance and candidate gene location

Association analysis of all the variants with phenotype information in the mapping population was performed using GLM (general linear model) model in TASSELE5.0. The target interval was defined as the two-side flanking regions (400 Kb distance) of the SNP/InDel variant with the lowest association *P* value. Distribution of the genome scaffolds in 13 chromosomes was numbered according to the chromosome annotation described by Zhao et al. [25]. Home-made scripts were used to screen the specific SNPs and InDels of the target interval. The genomic variants data of 822 sesame accessions (partially listed in Additional file 2: Table S2 at www.sesamum.org) and the SNP/InDel database of the population BSA pool (uploaded to NCBI database) of *cl* genotype were applied for the target variant detection.

The candidate SNP/InDel sites were transformed into PCR-based markers using the Primer Premier 5.0 program (http://www.premierbiosoft.com/products/products.html) according to the method of Wei et al. [41]. PCR reaction was performed in a 10 μL reaction mixture containing 1 × Buffer, 2.0 mmol L^− 1^ MgCl_2_, 0.1 mmol L^− 1^ dNTPs, 1 μmol L^− 1^ of each primer, 0.5 U Taq polymerase (Takara, Dalian, China), and 80 ng template DNA. Amplification was performed on a PTC-225 machine (MJ Research, Waltham, MA) using the following conditions: 94 °C for 3 min, 30 cycles of 30s at 94 °C, 30s at 55 °C, and 30s at 72 °C, with a final 6 min extension at 72 °C. All the PCR products were electrophoresed in 8% non-denaturing polyacrylamide gels and visualized via silver staining [42].

### Cloning and annotation of *Sicl1* gene

To clone the gDNA and cDNA sequences of *SiCL1* alleles in mutant *cl1* and the wild genotype, the primer pairs were designed using Primer Premier 5.0 (Additional file 5: Table S5, Additional file 7: Table S6). Total RNA was extracted from tissues using Trizol Reagent (Ambion, Life Technologies, USA) following the manufacturer’s instructions. First-strand cDNA synthesis was performed using RevertAid First Strand cDNA Synthesis Kit (Thermo Scientific, Germany) according to the manufacturer’s instruction. PCR was performed in a 50 μL reaction mixture containing 1 × Buffer, 2.0 mmol·L^− 1^ MgCl_2_, 0.1 mmol·L^− 1^ dNTPs, 1 μmol·L^− 1^ of each primer, 1.0 U Taq polymerase and 100~ 200 ng template DNA or 5 × diluting cDNA. Standard PCR reactions were carried out on a Eppendorf Mastercycler (Eppendorf, Germany) under the following conditions: 94 °C for 4 min; 35 cycles of 30s at 94 °C, 2 min at 55–58 °C and 1 min at 72 °C, with a final 8 min extension at 72 °C. PCR products were individually gel purified for Sanger sequencing. Non- redundant (NR) protein and Kyoto Encyclopedia of Genes and Genomes (KEGG) annotations for candidate genes in the interval were obtained using BLASTP and BLAST2GO, respectively (http://www.genome.jp/kegg/pathway.html).

### *Sicl1* homolog detection and phylogenetic analysis

BLASTN was applied to screen *SiCL1* homolog(s) in Yuzhi 11 reference genome. Gene alleles in sesame accessions were screened using MEGA 5.2 according to the Muscle method [43].

The amino acid sequences of *SiCL1* and *Sicl1* were aligned with the homologs in *Arabidopsis thaliana*, *Zea mays*, *Oryza sativa*, *Vitis vinifera*, *Manihot esculenta*, *Nicotiana tabacum*, *Hevea brasiliensis*, *Ipomoea nil*, *Coffea canephora*, and *Solanum tuberosum*, respectively, using DNAMAN (http://www.lynnon.com/pc/framepc.html). All the above homologs information was downloaded from NCBI dataset. A neighbor joining phylogenetic tree was constructed based on the above orthologs using the MEGA 5.2 program [43].

### RNA extraction and expression profile assay of *SiCL1* allele

Total RNA was extracted from tissues using Trizol Reagent (Ambion, Life Technologies, USA) following the manufacturer’s instructions. The primer pairs for quantitative real time PCR (qRT-PCR) analyses were designed with Primer Premier 5.0 program (http://www.premierbiosoft.com/products/products.html). The forward sequence (RT-CL1_F) was 5′CCTAACCCTCCATTCCCATT 3′ (Tm = 58.0 °C), and the reverse sequence (RT-CL1_R) was 5′GATACGACGACAGCCCACTAA 3′ (Tm = 57.8 °C). The amplicon size was 271 bp. Real-time PCR reaction was performed on a Mastercycler realplex (Eppendorf, Germany). The sesame *β-tubulin* gene was used as an endogenous reference gene [5]. Transcript amount of target genes was normalized against the *β-tubulin* gene and analyzed using ∆∆Ct method according to Wei et al. [44].

## Additional files

Additional file 1: **Table S1** Genomic variants screening for the curly leaf trait using genome variants data of sesame accessions. (XLSX 14 kb)

Additional file 2: **Table S2.** Variants of the candidate interval C29 linked to the leaf curling trait in 822 sesame accessions with normal leaf shape and a *cl1* mutant line (www.sesamum.org). (XLSX 14 kb)

Additional file 3: **Table S3.** Genomic variants screening for the curly leaf trait in sesame using BSA pool data. (XLSX 50 kb)

Additional file 4: **Table S4** Genome information of the six candidate variants for the curly leaf trait. *F refers to forward direction. R refers to reverse direction. - indicates that no genes are found in the 10 Kb flanking region of the variant site. **KEGG refers to Kyoto Encyclopedia of Genes and Genomes. C29.460 is the retained gene linked to the curly leaf trait in the interval C29 in this study. (XLSX 11 kb)

Additional file 5: **Table S5.** Information of primer pairs of the candidate markers for curly leaf trait. (XLSX 10 kb)

Additional file 6: **Figure S1.** Amplification of the *SiCLInDel 1* marker in the test F_2_ population and sesame germplasm. **M:** DNA marker; **Lane 1–10:** F_2_ individuals with curly leaf phenotype; **Lane 11–20:** F_2_ individuals with normal leaf phenotype; **Lane 21–40:** sesame germplasm materials (M1-M20) with normal leaf phenotype. (TIF 396 kb)

Additional file 7: **Table S6.** Information of primer pairs of the cDNA sequences of *SiCL1* alleles in sesame. Genome information of the six candidate variants for the curly leaf trait. Note: The italic letters in the sequences are the 5′ and 3′ UTR regions of *SiCL1* alleles. (XLSX 13 kb)

## Abbreviations

AFLP: Amplified fragment length polymorphisms
BSA: Bulked segregation analysis
CL: Curly leaf
DAP: Day after pollination
FAA: Formalin-glacial acetic acid-alcohol
GWAS: Genome wide association study
InDel: Insertion-deletion
LG: Linkage group
PC (ab): Abaxial parenchyma cell
PC (ad): Adaxial parenchyma cell
QTL: Quantitative trait loci
SNP: Single nucleotide polymorphism
