# Supplementary figures and images for: Identification of a SiCL1 gene controlling leaf curling and capsule indehiscence in sesame via cross-population association mapping and genomic variants screening
Source: BMC Plant Biol. 2018 Nov 22;18:296. doi: 10.1186/s12870-018-1503-2 (PMC6251216; doi:10.1186/s12870-018-1503-2)

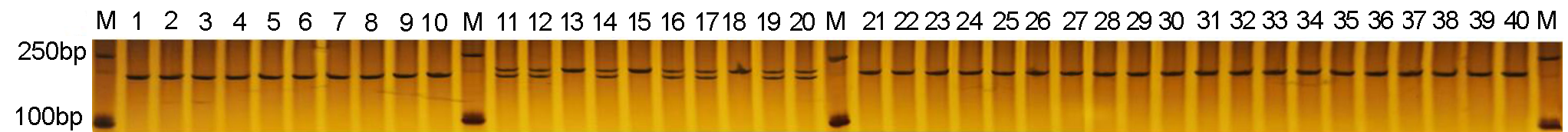

Supplement: Supplementary file 6 — Figure S1. Amplification of the SiCLInDel 1 marker in the test F2 population and sesame germplasm. M: DNA marker; Lane 1–10: F2 individuals with curly leaf phenotype; Lane 11–20: F2 individuals with normal leaf phenotype; Lane 21–40: sesame germplasm materials (M1-M20) with normal leaf phenotype. (TIF 396 kb) [file 12870_2018_1503_MOESM6_ESM.tif]
